# Association between the Fatty Liver Index and Risk of Type 2 Diabetes in the EPIC-Potsdam Study

**DOI:** 10.1371/journal.pone.0124749

**Published:** 2015-04-22

**Authors:** Susanne Jäger, Simone Jacobs, Janine Kröger, Norbert Stefan, Andreas Fritsche, Cornelia Weikert, Heiner Boeing, Matthias B. Schulze

**Affiliations:** 1 Department of Molecular Epidemiology, German Institute of Human Nutrition Potsdam-Rehbruecke, Nuthetal, Germany; 2 German Center for Diabetes Research (DZD), Neuherberg, Germany; 3 Department of Internal Medicine, Division of Endocrinology, Diabetology, Nephrology, Vascular Disease and Clinical Chemistry, University Hospital Tübingen, Tübingen, Germany; 4 Research Group Cardiovascular Epidemiology, German Institute of Human Nutrition Potsdam-Rehbruecke, Nuthetal, Germany; 5 Institute for Social Medicine, Epidemiology and Health Economics, Charité University Medical Center, Berlin, Germany; 6 Department of Epidemiology, German Institute of Human Nutrition Potsdam-Rehbruecke, Nuthetal, Germany

## Abstract

The fatty liver index (FLI) predicts fatty liver by using BMI, waist circumference, γ-glutamyltransferase and triglycerides. We investigated the association between the FLI and the risk of type 2 diabetes and evaluated to what extent single FLI components contribute to the diabetes risk. We analysed a case-cohort study (random sub-cohort: 1922; incident cases: 563) nested within the European Prospective Investigation into Cancer and Nutrition (EPIC)-Potsdam study. The proportion of exposure effect (PEE) explained by single FLI components was evaluated and effect decomposition using inverse probability weighting (IPW) was applied. Women and men with a FLI ≥60 compared to those with a FLI <30 had a multivariable-adjusted Hazard Ratio (HR) of 17.6; 95% confidence interval (CI) 11.1-28.0 and HR: 10.9; 95% CI 6.22-19.2, respectively. Adjustment for BMI or waist circumference attenuated this association in men [PEE_BMI _(95% CI) = 53.8% (43.9%-65.8%); PEE_waist _(95% CI) = 54.8% (44.2%-68.8%)]. In women, adjustment for waist circumference attenuated the association to a lesser degree than in men [PEE_waist _(95% CI) = 31.1%; (21.9%-43.1%)] while BMI had no appreciable effect [PEE_BMI _(95% CI) = 11.0% (2.68%-21.0%)]. γ-glutamyltransferase and triglycerides showed only a small attenuation in women [PEE_GGT_(95% CI) = 3.11% (-0.72%-4.48%); PEE_TG _(95% CI) = 6.36% (3.81%-9.92%)] and in men [PEE_GGT_ = 0%; PEE_TG _(95% CI) = 6.23% (2.03%-11.8%)]. In women, the total effect was decomposed into a direct effect and 4 indirect effects (HR_BMI _= 1.10; HR_waist_ = 1.28; HR_GGT_ = 0.97 and HR_TG _= 1.03). In men, the 4 indirect effects were HR_BMI _= 1.25; HR_waist_ = 1.29; HR_GGT_ = 0.97 and HR_TG _= 0.99. These data suggest that the FLI, as a proxy for fatty liver, is associated with risk of type 2 diabetes. This association is only partly explained by standard estimates of overall and abdominal body fatness, particularly among women.

## Introduction

Non-alcoholic fatty liver disease (NAFLD) is characterized by increased fat storage in form of triglycerides in the liver (exceeding 5% of its weight) and the absence of excessive alcohol consumption [1]. NAFLD is strongly linked to obesity, dyslipidemia, and insulin resistance and is therefore considered as the hepatic manifestation of metabolic syndrome [2]. In a large case-control study, patients suffering from type 2 diabetes had 80% more liver fat than age-, sex- and body weight-matched controls [3]. Furthermore, changes in liver fat content might modify the risk of type 2 diabetes. Within this context, the progression of fatty liver during a 5-year follow-up was associated with an increased risk of type 2 diabetes—independent of baseline BMI, changes in BMI over time as well as baseline blood glucose and insulin [4].

The gold standard for the diagnosis of NAFLD is liver biopsy [5]. However, this procedure is not suitable in all NAFLD-patients and its usage can lead to complications [6]. Therefore, non-invasive techniques like magnetic resonance spectroscopy (MRS), computed tomography (CT) and ultrasound are often used for diagnosing fatty liver in epidemiological studies. While MRS is very sensitive in detecting lower degrees of hepatic fat, ultrasound and CT provide rather semi-quantitative measurements [7]. Furthermore, these methods are associated with high costs and are therefore usually not suitable in a large population-based screening of fatty liver. Thus, alternative estimates of NAFLD based on routine risk factors have been proposed. For example, Bedogni et al. developed a simple algorithm for predicting fatty liver based on ultrasonography in 496 individuals in 2006 [8]. The fatty liver index (FLI) consists of body mass index (BMI), waist circumference, γ-glutamyltransferase (GGT) and triglycerides. This index was validated against MRS in a German population and provided moderate diagnostic accuracy [area under the receiver operating characteristic curve (AUROC) = 0.72; 95% CI 0.59–0.92] [9]. Up to now, only two previous studies evaluated the association between FLI and incident diabetes, in a French and a Korean population [10, 11]. However, the FLI includes BMI and waist circumference in its computation, two major anthropometric risk factors of type 2 diabetes, and it remains unclear to which extent single components of the FLI contribute to the risk association and whether the FLI predicts diabetes beyond routine measures of body fat.

Therefore, the aim of this study was to evaluate whether fatty liver estimated by FLI is associated with risk of type 2 diabetes within the EPIC-Potsdam study. Furthermore, we determined to what extent this association is attributable to its single components.

## Methods

### Study population

The European Prospective Investigation into Cancer and Nutrition (EPIC)-Potsdam study consists of 27,548 participants (16,644 women and 10,904 men) recruited between 1994 and 1998 from the general population in Potsdam and surroundings. The baseline examination involved a personal interview including questions on prevalent diseases, a self-administered questionnaire on socio-economic and lifestyle characteristics, interviewer-conducted anthropometric measurements and a blood sample collection [12, 13]. To evaluate biochemical risk factors for type 2 diabetes, a prospective case-cohort study within the EPIC-Potsdam study was designed, described in detail previously [14]. From 26,444 participants who provided blood samples at baseline, 2,500 individuals were randomly selected. We excluded individuals with liver cancer (n = 1), liver medication (n = 7) and medication for treating hyperlipidemia (n = 187), as well as participants with missing data on biomarker measurements (n = 356). Further exclusion criteria were implausible energy intake (<800 or >6000 kcal/day), prevalent diabetes and participants with uncompleted follow-up questionnaires, leaving 1922 individuals (1215 women and 707 men) for analyses in the sub-cohort. Identical exclusion criteria were applied for type 2 diabetes cases, leaving 563 incident cases (224 women and 339 men; overlap of 24 women and 33 men within the sub-cohort due to the case-cohort design).

### Ethics statement

All participants provided written informed consent and permission was given by the ethics committee of the State of Brandenburg, Germany.

### FLI as a surrogate measure of fatty liver

The FLI was developed in an Italian population aged 18 to 75 years old (61% males) including 216 individuals with and 280 individuals without suspected liver disease [8]. Within this population, the presence of fatty liver was identified by using ultrasound. Bedogni et al. used bootstrapped stepwise logistic regression to derive the final score [8]. Out of 13 variables (including gender, age, ethanol intake, alanine transaminase, aspartate transaminase, GGT, BMI, waist circumference, sum of 4 skinfolds, glucose, insulin, triglycerides and cholesterol) four predictors remained within the score:

$$FLI=\left(e^{\left(0.953\times ln\left(TG\right)+0.139\times BMI+0.718\times ln\left(GGT\right)+0.053\times WC-15.745\right)}\right)/\left(1+e^{\left(0.953\times ln\left(TG\right)+0.139\times BMI+0.718\times ln\left(GGT\right)+0.053\times WC-15.745\right)}\right)\times 100$$

TG: triglycerides in mg/dl
GGT: γ-glutamyltransferase in U/l
WC: waist circumference in cm

The FLI ranges from 0 to 100 and in the population of Bedogni et al. a FLI <30 ruled out and a FLI ≥60 ruled in fatty liver with a good diagnostic accuracy (AUROC = 0.85; 95% CI 0.81–0.88).

### Assessment of covariates

Socio-economic and lifestyle factors such as level of education, occupation, smoking behavior and physical activity were assessed by a self-administered questionnaire and a personal interview. Weight, height and waist circumference were measured at baseline by trained interviewers and followed standard protocols under strict quality control [15]. Body weight was measured by electronic digital scales, accurate to 0.1 kg (Soehnle, type 7720/23, Murrhardt, Germany). Individuals wore only light underwear and had emptied their bladder. Heights were measured to the nearest 0.1 cm using a flexible anthropometer. Waist circumference was obtained in the standing position with weight distributed equally over both feet. Waist circumference was taken to the nearest 0.5 cm and measured with a non-stretching tape applied horizontally midway between the lower rib margin and the superior anterior iliac spine [16]. Dietary intake during the last twelve months was assessed through a validated food frequency questionnaire (FFQ) [17].

### Determination of biomarkers

Biomarker measurement was conducted in blood samples stored at -80°C or lower until analysis. Plasma levels of C-reactive protein (CRP), triglycerides, blood glucose, HDL-cholesterol, total cholesterol, GGT, alanine transaminase (ALT), fetuin-A and erythrocyte levels of HbA_1c_ were measured with the automatic ADVIA 1650 analyzer (Siemens Medical Solutions, Erlangen, Germany) [14, 18]. For determination of fetuin-A, an immunoturbidimetric method was used with specific polyclonal goat anti-human fetuin-A antibodies to human fetuin-A (BioVendor Laboratory Medicine, Modreci, Czech Republic) [14]. Plasma adiponectin concentrations were determined through ELISA (Linco Research, St. Charles, MO, USA) [19]. All assay procedures were performed according to the manufacturer’s description. LDL-cholesterol levels were calculated using the Friedewald equation [20].

Blood samples were drawn in monovettes containing citrate as anticoagulant. Therefore plasma concentrations were multiplied by 1.16 for women and 1.17 for men in order to receive levels for these citrate plasma samples comparable to levels obtainable from EDTA plasma [21].

### Assessment of type 2 diabetes

Every 2–3 years follow-up questionnaires were sent out to identify incident cases of diabetes, with response rates of >90% [22]. Incident type 2 diabetes was evaluated by a physician with information from self-reported medical diagnoses, medication records, and dieting behavior. Furthermore, additional information from death certificates or from random sources, such as a tumor center, physician, or clinic, which provided assessments from other diagnoses, was obtained. Although the self-reports of type 2 diabetes were generally reliable [23], all incident cases were verified by treating physicians. They were asked to provide data on the date and type of diagnosis, on diagnostic tests and the treatment in a questionnaire. Cases confirmed by a physician (International Classification of Diseases, 10^th^ Edition (ICD10): E11) and a diagnosis date after the baseline examination were considered as confirmed incident cases of type 2 diabetes. For the current analysis, we used data collected until the end of the fourth follow-up period in August 2005.

### Statistical analysis

Associations between the FLI and diabetes risk were evaluated using Cox regression modified for the case-cohort design according to the Prentice method [24]. The proportional hazards assumption was tested via plotting of the Schönfeld residuals [25]. Age was used as the underlying time scale in all models, with entry time defined as the participant’s age at recruitment and exit time defined as the age at the end of follow-up based on the date of diagnosis, death, or return of the last follow-up questionnaire. The analysis was stratified for age at the baseline examination in one-year intervals. Cox models were further adjusted for education (no vocational training or in training, vocational training, technical school, technical college or university), occupation (sedentary, standing, (heavy) manual work), smoking behavior (never smoker, ex-smoker, current smoker <20 units/day, current smoker ≥20 units/day), sport activities (no sport, ≤4 h/week, >4 h/week), biking (no biking, <2.5 h/week, 2.5–4.9 h/week, ≥5 h/week), alcohol intake (no alcohol intake, >0–6 g/day; >6–12 g/day; >12–24 g/day; >24–60 g/day; >60–96 g/day; >96 g/day), coffee consumption (ml/day), red meat intake (g/day) and intake of whole-grain bread (g/day). In sensitivity analyses, individuals with a daily alcohol consumption of >30 g for men and >20 g for women were excluded [1]. Furthermore, we evaluated whether lifetime alcohol intake has an influence by excluding participants who were former heavy drinkers or consumed alcohol occasionally heavy or always heavy during their lifetime [26]. In a third sensitivity analyses the study population was restricted to participants who had fasted for at least eight hours or had not eaten but drunk within the past eight hours. Finally, participants in a prediabetic state (HbA_1c_ ≥5.7) were excluded.

To evaluate the impact of single components within the FLI, the Cox models were further adjusted for BMI, waist circumference, GGT or triglycerides at baseline. The proportion of exposure effect (PEE) [27] explained by FLI components was calculated:

$$PEE=\left[1-\left(\beta_{adjusted}/\beta_{crude}\right)\right]\times 100$$

95% confidence intervals (CI) of PEE were estimated by bootstrapping method with a sampling rate of 80% and 1000 replicates which was adapted for case-cohort design [28]. Furthermore, differences in β-coefficients with and without adjustment for anthropometry were tested one-tailed for statistical significance (according to [29]).

Mediation analysis was performed using the approach introduced by Lange et al. [30, 31]. This method relies on the counterfactual framework of mediation analysis [32] and provides the possibility to decompose the total effect of a given exposure X on the outcome Y into a natural direct effect (X→Y) and a natural indirect effect through each mediator M (X→M→Y) by using inverse probability weighting (IPW). In the present analysis, we considered the four FLI components (BMI, waist circumference, GGT and triglycerides) as mediators of the association between the FLI and risk of type 2 diabetes. However, one important assumption is that the mediators should be independent from each other. This is difficult to fulfill when investigating highly correlated factors such as BMI and waist circumference. Therefore, we further transformed waist circumference by adding up the median waist circumference with the residuals obtained from a linear regression of waist circumference and BMI adjusted for the same covariates, used within the Cox regression. 95% CI for direct and indirect effects were obtained by using a sampling rate of 80% and 1000 bootstrap replicates applied for case-cohort design [28].

All data analyses were performed by using the software package Statistical Analysis System (SAS) Enterprise Guide 6.1 with SAS version 9.4 (SAS Institute Inc., Cary, NC, USA). We considered *p*<0.05 as being statistically significant.

## Results

Table 1 represents the baseline characteristics for women and men according to categories of the FLI. In higher FLI categories women were less educated and slightly more likely to perform standing occupation or heavy manual work. However, women were less physically active during their leisure time. There was no clear association between the FLI and smoking status in women. Women in higher FLI categories consumed less alcohol. Men in the highest FLI category were slightly older, less educated and more likely to be an ex-smoker. They were less physically active, consumed more red meat and less whole-grain bread and coffee. Contrary to women, the alcohol consumption increased with higher FLI in men. BMI and waist circumference were positively associated with the FLI in women and men.

**Table 1:** Baseline characteristics by categories of the fatty liver index in EPIC-Potsdam^a^.

	FLI categories women			FLI categories men		
	<30	30-<60	≥60	<30	30-<60	≥60
FLI, median (IQR)	7.17 (10.4)	42.5 (15.2)	77.0 (22.0)	16.8 (14.5)	44.0 (13.5)	78.2 (19.1)
**n**	878	175	162	201	222	284
age at baseline, median (IQR)	45.0 (15.0)	55.0 (14.0)	53.0 (16.0)	51.0 (14.0)	51.0 (15.0)	52.0 (14.0)
**Education, %**						
no vocational training/ in training/ vocational training	36.2	51.4	50.6	25.4	35.1	33.8
technical school	29.8	22.9	33.3	15.9	12.6	19.4
technical college or university	33.9	25.7	16.1	58.7	52.3	46.8
**Occupation, %**						
sedentary occupation	64.6	58.3	55.6	58.2	55.0	55.6
standing occupation	32.5	38.9	38.9	32.8	32.0	32.0
(heavy) manual work	2.96	2.86	5.56	8.96	13.1	12.3
**Smoking status, %**						
never smoker	58.5	55.4	54.9	38.3	33.8	24.7
ex-smoker	23.9	25.1	29.0	34.3	40.5	50.7
smoker <20 units/day	14.6	13.1	13.6	16.4	13.5	15.9
smoker ≥20 units/day	2.96	6.29	2.47	11.0	12.2	8.80
physical activity, median (IQR)^b^	2.00 (4.00)	1.50 (3.50)	1.00 (3.00)	2.00 (4.50)	2.00 (4.00)	1.00 (3.75)
alcohol intake in g/day, median (IQR)	5.52 (8.91)	4.63 (9.62)	4.23 (8.10)	14.8 (20.9)	15.8 (19.3)	20.9 (32.0)
red meat intake in g/day, median (IQR)	30.7 (25.9)	32.2 (24.6)	31.9 (29.1)	41.2 (29.9)	48.0 (39.4)	51.9 (39.3)
whole-grain bread intake in g/day, median (IQR)	31.1 (62.8)	31.4 (57.8)	28.7 (62.6)	23.1 (54.9)	14.1 (50.0)	16.0 (52.3)
coffee intake in g/day, median (IQR)	300 (300)	450 (300)	300 (450)	450 (386)	450 (300)	300 (450)
BMI in kg/m², median (IQR)	23.4 (3.85)	28.2 (3.26)	32.7 (5.30)	23.6 (2.63)	26.0 (2.52)	29.1 (3.47)
waist circumference in cm, median (IQR)	74.5 (10.0)	88.0 (7.40)	99.0 (12.5)	85.0 (8.00)	92.0 (7.00)	101 (10.3)
**Biomarker**						
HDL-cholesterol in mmol/l, median (IQR)	1.58 (0.48)	1.45 (0.43)	1.32 (0.31)	1.42 (0.47)	1.26 (0.39)	1.18 (0.33)
LDL-cholesterol in mmol/l, mean (SD)	2.92 (0.85)	3.33 (0.98)	3.25 (0.86)	3.12 (0.88)	3.33 (0.83)	3.26 (0.97)
Triglycerides in mmol/l, median (IQR)	0.92 (0.54)	1.43 (0.80)	1.82 (1.26)	0.98 (0.57)	1.44 (0.91)	2.26 (1.55)
Total Cholesterol in mmol/l, mean (SD)	4.98 (1.04)	5.58 (1.13)	5.57 (1.05)	5.01 (1.05)	5.39 (1.00)	5.69 (1.08)
ALT in U/l, median (IQR)	15.1 (5.80)	20.9 (11.6)	24.4 (12.8)	19.9 (8.19)	25.7 (14.0)	35.1 (21.1)
GGT in U/l, median (IQR)	11.6 (6.96)	19.7 (15.1)	27.3 (27.8)	15.2 (10.5)	26.9 (23.4)	43.3 (38.0)
ALT in μkat/l, median (IQR)	0.25 (0.10)	0.35 (0.19)	0.41 (0.21)	0.33 (0.14)	0.43 (0.23)	0.59 (0.35)
GGT in μkat /l, median (IQR)	0.19 (0.12)	0.33 (0.25)	0.46 (0.46)	0.25 (0.18)	0.45 (0.39)	0.72 (0.64)
Fetuin-A in μg/ml, mean (SD)	269 (63.6)	274 (61.9)	280 (62.3)	257 (57.6)	264 (55.2)	264 (52.5)
CRP in nmol/l, median (IQR)	4.42 (11.1)	15.5 (30.9)	30.9 (44.2)	2.23 (6.69)	5.57 (11.1)	8.91 (15.6)
Adiponectin in μg/ml, median (IQR)	9.69 (5.13)	8.23 (4.85)	7.29 (3.90)	7.20 (3.92)	6.04 (3.13)	5.38 (2.94)
Glucose in mmol/l, median (IQR)	5.39 (0.79)	5.64 (0.97)	5.70 (0.91)	5.53 (0.86)	5.80 (0.95)	5.93 (1.08)
HbA1c in mmol/mol, median (IQR)	33.7 (5.79)	35.7 (7.32)	37.5 (7.32)	34.1 (5.68)	35.7 (6.78)	36.3 (8.03)

IQR, interquartile range; FLI, fatty liver index; ALT, alanine transaminase; GGT, γ-glutamyltransferase; CRP, C-reactive protein

^a^ women: n = 1215; men: n = 707

^b^ Sum of biking and sporting activities in h/week

Markers of dyslipidemia such as triglycerides and total cholesterol increased with higher FLI, whereas HDL-cholesterol was inversely associated with the FLI. Liver enzymes (ALT, GGT), fetuin-A and CRP increased with higher FLI. Adiponectin showed an inverse association with higher FLI, whereas blood glucose and HbA_1c_ showed slightly positive trends.

Hazard ratios (HR) for type 2 diabetes stratified by sex according to categories of the FLI are shown in Table 2. Compared to women in the reference category, women in the top third FLI category (≥60) had a HR of 17.1 (95% CI 11.3–25.9) in the age-stratified model. Adjustment for lifestyle and other socioeconomic factors did not affect this association: HR 17.6 (95% CI 11.1–28.0).

**Table 2:** HR (95% CI) for type 2 diabetes by categories of the fatty liver index in EPIC-Potsdam^a^.

	FLI categories women			FLI categories men		
	<30	30-<60	≥60	<30	30-<60	≥60
**FLI, median (IQR)** ^b^	7.17 (10.4)	42.5 (15.2)	77.0 (22.0)	16.8 (14.5)	44.0 (13.5)	78.2 (19.1)
**n (cases)**	39	71	138	17	62	293
**Model 1** (age-stratified)	1	7.03 (4.50–11.0)	17.1 (11.3–25.9)	1	3.05 (1.71–5.44)	11.4 (6.70–19.3)
**Model 2** (multivariable-adjusted)	1	7.25 (4.51–11.7)	17.6 (11.1–28.0)	1	2.53 (1.37–4.67)	10.9 (6.22–19.2)
**Model 2 + BMI**	1	6.24 (3.75–10.4)	12.8 (7.15–23.1)	1	1.58 (0.84–2.95)	3.03 (1.62–5.67)
*PEE*		7.54% (1.88%–15.1%)*	11.0% (2.68%–21.0%)*		51.2% (33.2%–78.6%)*	53.8% (43.9%–65.8%)*
**Model 2 + waist circumference**	1	4.78 (2.83–8.06)	7.22 (3.76–13.9)	1	1.53 (0.82–2.86)	2.95 (1.57–5.55)
*PEE*		21.0% (14.4%–30.6%)*	31.1% (21.9%–43.1%)*		54.2% (35.0%–85.5%)*	54.8% (44.2%–68.8%)*
**Model 2 + BMI, waist circumference**	1	4.86 (2.88–8.20)	7.63 (3.98–14.6)	1	1.49 (0.80–2.80)	2.67 (1.42–5.05)
*PEE*		20.2% (13.6%–29.4%)*	29.2% (19.5%–41.3%)*		56.9% (37.3%–89.2%)*	58.9% (47.7%–73.0%)*
**Model 2 + GGT**	1	6.98 (4.34–11.2)	16.1 (10.1–25.8)	1	2.53 (1.37–4.67)	10.9 (6.18–19.2)
*PEE*		1.84% (-0.43%–2.80%)*	3.11% (-0.72%–4.48%)*		*0%*	*0%*
**Model 2 + triglycerides**	1	6.43 (3.92–10.5)	14.7 (8.95–24.1)	1	2.40 (1.30–4.45)	9.42 (5.22–17.0)
*PEE*		6.03% (3.44%–9.34%)*	6.36% (3.81%–9.92%)*		*5*.*62% (1*.*54%–13*.*4%)*	6.23% (2.03%–11.8%)*
**Model 2 + GGT, triglycerides**	1	6.18 (3.77–10.1)	13.3 (8.02–21.9)	1	2.40 (1.30–4.45)	9.44 (5.22–17.0)
*PEE*		8.01% (4.54%–11.3%)*	9.92% (5.56%–14.0%)*		*5*.*53% (1*.*53%–14*.*8%)*	6.15% (1.84%–13.1%)*
**Model 2 + BMI, waist, GGT, triglycerides**	1	3.60 (2.09–6.18)	4.09 (2.01–8.31)	1	1.39 (0.74–2.61)	2.16 (1.10–4.22)
*PEE*		35.3% (26.7%–46.9%)*	50.9% (38.6%–66.1%)*		64.5% (42.2%–101%)*	67.9% (55.3%–85.6%)*

FLI, fatty liver index; PEE, Proportion of exposure effect

^a^ women: n = 1439, men: n = 1046

^b^ in sub-cohort

* significant change in β-coefficients compared to the multivariable-adjusted model

Model 2 is further adjusted for education (no vocational training or in training, vocational training, technical school, technical college or university), occupation (sedentary, standing, (heavy) manual work), smoking behavior (never smoker, ex-smoker, current smoker <20 units/day, current smoker ≥20 units/day), sport activities (no sport, ≤4 h/week, >4 h/week), biking (no biking, <2.5 h/week, 2.5–4.9 h/week, ≥5 h/week), alcohol intake (women: no alcohol intake, >0–6 g/day, >6–12 g/day, >12–24 g/day, >24–60 g/day, >60 g/day; men: no alcohol intake, >0–6 g/day, >6–12 g/day, >12–24 g/day, >24–60 g/day, >60–96 g/day, >96 g/day), coffee consumption (ml/day), red meat intake (g/day), intake of whole-grain bread (g/day).

Adjustment for BMI or waist circumference at baseline led to an attenuation of the association between the FLI and risk of type 2 diabetes in women and men. In women, the strongest attenuation was observed for waist circumference in the highest category [PEE_waist_ (95% CI) = 31.1% (21.9%–43.1%)], whereas BMI adjustment had only a small effect [PEE_BMI_ (95% CI) = 11.0% (2.68%–21.0%)]. Inclusion of BMI and waist circumference simultaneously resulted in a similar attenuation of the association as waist circumference alone [PEE_BMI + waist_ = 29.2% (19.5%–41.3%)]. The inclusion of GGT or triglycerides within the model led to a weaker attenuation [PEE_GGT_ (95% CI) = 3.11% (-0.72%–4.48%); PEE_TG_ (95% CI) = 6.36% (3.81%–9.92%)].

In the age-stratified model, men in the third category (≥60) had a HR of 11.4 (95% CI 6.70–19.3) (see Table 2). Adjustment for lifestyle and other socioeconomic factors showed no substantial change: HR 10.9 (95% CI 6.22–19.2). Further adjustment for BMI or waist circumference at baseline attenuated the association between the FLI and risk of type 2 diabetes in men, and no differences between both measures were observed [PEE_BMI_ (95% CI) = 53.8% (43.9%–65.8%) vs. PEE_waist_ (95% CI) = 54.8% (44.2%–68.8%)]. Adjustment for GGT showed no attenuating effect in men. The inclusion of triglycerides resulted in a PEE_TG_ (95% CI) = 6.23 (2.03%–11.8%) in the highest category.

In sensitivity analyses, the exclusion of non-fasted or prediabetic participants or participants with moderate or high baseline alcohol consumption or with higher lifetime alcohol consumption did not affect the overall results (S1–S4 Tables).

Table 3 illustrates the results of the effect decomposition of the association between the FLI and the type 2 diabetes risk by using the IPW-method. In women, the total HR of 3.00 was decomposed into a direct HR for the FLI of 2.13 and an indirect HR of 1.41 which was decomposed into HR_BMI_ = 1.10; HR_waist_ = 1.28; HR_GGT_ = 0.97 and HR_TG_ = 1.03. In men, the indirect HR was decomposed into HR_BMI_ = 1.25; HR_waist_ = 1.29; HR_GGT_ = 0.97 and HR_TG_ = 0.99, which resulted in a total effect of 2.22×1.25×1.29×0.97×0.99 = 3.44. When waist circumference was adjusted for BMI, it still explained a small proportion of the total effect in women (5.73%) whereas in men, there was no effect HR_waist transformed_ = 0.99.

**Table 3:** Total, direct and indirect effects with 95% CI of the association between the fatty liver index and the risk of type 2 diabetes within EPIC-Potsdam^a^.

	waist circumference untransformed				waist circumference transformed			
	women		men		women		men	
	HR (95% CI)	% of the total effect	HR (95% CI)	% of the total effect	HR (95% CI)	% of the total effect	HR (95% CI)	% of the total effect
**direct effect**	2.13 (1.43–3.19)	68.9	2.22 (1.75–2.81)	64.5	2.66 (2.09–3.38)	77.2	2.50 (2.11–2.94)	74.5
**indirect effect**	1.41 (1.04–1.90)	31.1	1.55 (1.34–1.80)	35.5	1.33 (1.20–1.48)	22.8	1.37 (1.28–1.46)	25.5
**total effect**	3.00		3.44		3.55		3.41	
**BMI**	1.10 (0.98–1.24)	8.92	1.25 (1.16–1.35)	17.9	1.26 (1.09–1.45)	18.0	1.38 (1.28–1.49)	26.3
**waist**	1.28 (1.02–1.62)	22.6	1.29 (1.15–1.45)	20.8	1.08 (1.03–1.12)	5.73	0.99 (0.96–1.02)	-0.93
**GGT**	0.97 (0.91–1.03)	-3.02	0.97 (0.95–1.00)	-2.17	0.97 (0.92–1.02)	-2.63	0.99 (0.98–1.00)	-0.85
**triglycerides**	1.03 (0.97–1.09)	2.62	0.99 (0.93–1.05)	-1.03	1.02 (0.95–1.10)	1.74	1.01 (0.97–1.06)	0.98

GGT, gamma-glutamyltransferase

^a^ women: n = 1439, men: n = 1046

The exposure was defined as FLI categories (<30, 30-<60, ≥60); waist circumference was transformed by adding up the median waist circumference with the residuals obtained from a linear regression of waist circumference and BMI which was adjusted for age at baseline, education (no vocational training or in training, vocational training, technical school, technical college or university), occupation (sedentary, standing, (heavy) manual work), smoking behavior (never smoker, ex-smoker, current smoker <20 units/day, current smoker ≥20 units/day), sport activities (no sport, ≤4 h/week, >4 h/week), biking (no biking, <2.5 h/week, 2.5–4.9 h/week, ≥5 h/week), alcohol intake (women: no alcohol intake, >0–6 g/day, >6–12 g/day, >12–24 g/day, >24–60 g/day, >60 g/day; men: no alcohol intake, >0–6 g/day, >6–12 g/day, >12–24 g/day, >24–60 g/day, >60–96 g/day, >96 g/day), coffee consumption (ml/day), red meat intake (g/day), intake of whole-grain bread (g/day).

## Discussion

In the present study, the FLI was associated with a higher risk of type 2 diabetes in women and men. This association was independent of potential confounding socioeconomic and lifestyle risk factors. Further adjustment for BMI or waist circumference attenuated this association in men. However, in women, adjustment for BMI had no significant effect and the inclusion of waist circumference only moderately attenuated the association. GGT and triglycerides explained only small proportions in both sexes.

Our results of a strong positive association between the FLI, as a surrogate measure for fatty liver, and type 2 diabetes risk are in line with other cohort studies on NAFLD and incident type 2 diabetes [4, 10, 33–43]. However, most research was conducted in Asian individuals undergoing periodic health examinations [4, 33, 35–40], and only one study focused on Caucasian (French volunteers) [10]. Similar to our results, Balkau et al. found a stronger risk relation in women compared to men, although it was significant in both sexes [10]. Studies among Japanese and Korean men were consistent with our finding, that fatty liver is an independent risk factor for type 2 diabetes in men [34, 43]. The remaining studies showed no sex-stratified estimates, however, they found fatty liver to be associated with increased risk of type 2 diabetes within their cohorts [4, 35–42].

The adjustment for potential confounders differed between the studies. While some adjusted for age, sex and lifestyle variables, others additionally included family history of diabetes or measures of insulin resistance and all studies found an independent association between NAFLD and incident type 2 diabetes. Contrary to the previous studies, we additionally adjusted for dietary risk factors for type 2 diabetes. However, these factors together with other confounding lifestyle and socioeconomic factors had only a slight impact on the observed association in our study. Alcohol consumption is an important factor to distinguish between NAFLD and alcoholic fatty liver disease. Most studies excluded participants with a higher alcohol intake and adjusted for alcohol consumption, while others only adjusted for alcohol intake—as we did in the main analysis. However, our sensitivity analyses, excluding participants with higher alcohol consumption at baseline or during lifetime yielded essentially similar results.

While most previous cohort studies used ultrasound to determine fatty liver, two previous studies used the FLI [10, 11]. However, both studies used different cut-off values when evaluating diabetes risk compared to our analysis. While we used the original categories suggested by Bedogni et al. [8], Balkau et al. [10] did not describe their decision for using FLI <20 and FLI ≥70 as cut-offs. Jung et al. [11] used a lower cut-off to rule out fatty liver based on a previous study which found that the likelihood not to have fatty liver was greater than 91% by using FLI <20 [44]. Nevertheless, when we applied these cut-offs to our data, the results were not markedly changed (data not shown).

In our study, the FLI was associated with diabetes risk even after adjustment for BMI and waist circumference—two major components of the index. This is generally in line with previous studies which observed significant relations of fatty liver with diabetes after adjustment for BMI [34, 35]. Two previous studies also considered waist circumference as covariate and—similar to our study—found a significant increased risk for type 2 diabetes among participants with fatty liver [41, 43]. Altogether, these findings are in agreement with data showing that liver fat content is associated with insulin resistance and prediabetes—stronger than total and visceral fat mass [45, 46].

We observed that single anthropometric FLI components seem to play a different role for diabetes risk in women and men. While in men further adjustment for either BMI or waist circumference revealed a similar strong attenuation of the risk association, in women, adjustment for BMI showed almost no effect and adjustment for waist circumference only moderately attenuated the association. The results obtained from the effect decomposition method using IPW agree with these findings. Overall this analysis suggests that the FLI provides additional information beyond waist circumference and BMI in women, whereas in men, the observed association with diabetes risk may mainly be driven by these two parameters. Contrary to our findings, studies using ultrasound to assess fatty liver did not observe different attenuation of the association with type 2 diabetes between the sexes when adjusting for BMI [33, 36].

Additionally, Feller et al. observed a negative interaction between waist circumference and BMI in EPIC-Potsdam [47] with weaker associations between waist circumference and risk of type 2 diabetes at higher BMI. However, the FLI does not include an interaction term of BMI and waist circumference; therefore, we did not model an interaction of both components in our analysis.

While adjustment for BMI and waist circumference led to stronger attenuations, adjustment for GGT and triglycerides—two established risk factors for type 2 diabetes—explained only small proportions of the diabetes risk. This might be due to the fact that in the study of Bedogni et al. [8] GGT and triglycerides were only minor predictors for fatty liver. Overall, the association between the FLI and risk of type 2 diabetes does not seem to be mediated by these two parameters in our study.

None of the previous studies using the FLI presented sex-stratified models with and without adjustment for its single components. Within this context, it is debatable whether the FLI formula is equally suitable for women and men. The FLI does not account for sex as predictive factor even though the prevalence of fatty liver is higher in men than in women [48, 49]. Bedogni et al. [8] used bootstrapped stepwise logistic regression and tested several models to derive the final score, including sex as predictor. However, only triglycerides, BMI, GGT and waist circumference remained as the most predictive factors, which might indicate that waist circumference captures most of the information provided by sex. Within the study of Bedogni et al. [8] 61% of the participants were men and fatty liver was less frequent among women (34% vs. 54%). Similarly, in our study population women were less likely to have fatty liver as predicted by the FLI. However, women with a FLI ≥60 showed high CRP levels and therefore might have a more progressive form of fatty liver [50] compared to the men. Consequently, sex-specific cut-offs to predict fatty liver might be reasonable.

Our study has several limitations. Although Bedogni et al. [8] used fasting blood samples to measure triglyceride levels and GGT, we included also non-fasted participants in our analysis. However, it has been shown that triglyceride levels increase only modestly in response to normal food intake in the general population [51]. Additionally, in our sensitivity analysis, the exclusion of non-fasted participants did not materially change the results.

Another limitation is that we could not differentiate between alcoholic fatty liver disease and non-alcoholic fatty liver disease in our study. Nevertheless, exclusion of participants consuming >20 g/day (women) or >30 g/day (men) did not affect the association. Because participants might have changed their alcohol intake before the baseline examination due to increased liver enzymes or other health problems, we accounted for lifetime alcohol consumption, while previous studies only considered alcohol consumption at baseline. Indicating robustness of our findings, we observed no substantial changes in our results.

A further limitation of our study refers to the usage of the FLI as a surrogate measure for fatty liver. In large epidemiological studies, techniques such as ultrasound or MRS are rarely available and expensive. The FLI provided a good diagnostic accuracy for fatty liver in the original study population (AUROC = 0.85; 95% CI 0.81–0.88). External validation studies in German and Korean individuals found comparable AUROC values by using ultrasound [52–55]. However, especially in validation studies against MRS [9] or liver biopsy [56, 57] lower AUROC values were obtained. Several factors such as NAFLD measurement and characteristics of the study population might have contributed to that. First, the FLI was developed by using ultrasound, thus validation studies using the same technique found a better diagnostic accuracy. Although ultrasonography is an accurate and reliable tool to detect moderate to severe fatty liver (≥20–30%), with a sensitivity of 84.8% and a specificity of 93.6% [58], the ability to detect lower percentages of liver fat by ultrasound might be limited [59, 60]. Second, the FLI was derived from a selected population in which 61.5% were male, 43.5% of the individuals had suspected liver disease and the average BMI was 27 kg/m^2^.

The strengths of our study include the prospective design, the wide assessment of potential confounding factors and the usage of validated type 2 diabetes cases. Although the FLI can only be interpreted as a surrogate measure for fatty liver, this index uses four variables which can easily be obtained and therefore, the FLI might be relevant for clinical practice. However, further investigation regarding its predictive power in women and men might be useful.

In conclusion, our data suggest that the FLI, as a proxy for fatty liver, is positively associated with risk of type 2 diabetes. This association is only partly explained by standard estimates of overall and abdominal body fatness, particularly among women.

## Supporting Information

S1 Table HR (95% CI) for type 2 diabetes in women (alcohol intake ≤20g/day) and men (alcohol intake ≤30g/day) by categories of the fatty liver index in EPIC-Potsdam. (DOCX)

S2 Table HR (95% CI) for type 2 diabetes by categories of the fatty liver index in EPIC-Potsdam after exclusion of participants with heavy lifetime alcohol consumption (former heavy drinkers, occasionally heavy drinkers, always heavy drinkers). (DOCX)

S3 Table HR (95% CI) for type 2 diabetes in fasting participants by categories of the fatty liver index in EPIC-Potsdam. (DOCX)

S4 Table HR (95% CI) for type 2 diabetes in participants without prediabetes (HbA1c <5.7) by categories of the fatty liver index in EPIC-Potsdam. (DOCX)
