# Supplementary material for: Association between the Fatty Liver Index and Risk of Type 2 Diabetes in the EPIC-Potsdam Study
Source: PLoS One. 2015 Apr 22;10(4):e0124749. doi: 10.1371/journal.pone.0124749 (PMC4406732; doi:10.1371/journal.pone.0124749)
Supplement: S1 Table — (DOCX) [file pone.0124749.s001.docx]

**S1_Table** HR (95% CI) for type 2 diabetes in women (alcohol intake ≤20g/day) and men (alcohol intake ≤30g/day) by categories of the fatty liver index in EPIC-Potsdam^a^

|  | **FLI categories women** | | |  | **FLI categories men** | | |
| --- | --- | --- | --- | --- | --- | --- | --- |
|  | **<30** | **30-<60** | **≥60** |  | **<30** | **30-<60** | **≥60** |
| **FLI, median (IQR)^b^** | 7.33 (10.6) | 42.3 (15.0) | 78.3 (21.8) |  | 17.2 (13.5) | 44.0 (14.0) | 78.2 (18.4) |
| **n (cases)** | 37 | 70 | 125 |  | 15 | 50 | 213 |
| **Model 1**  (age-stratified) | 1 | 7.61  (4.83-12.0) | 15.9  (10.4-24.4) |  | 1 | 2.69  (1.44-5.04) | 11.1  (6.26-19.5) |
| **Model 2**  (multivariable-adjusted) | 1 | 7.80  (4.83-12.6) | 15.8  (9.83-25.2) |  | 1 | 2.20  (1.11-4.36) | 10.2  (5.49-18.9) |

FLI, fatty liver index

^a^ women: n=1292, men: n=780; ^b^ in sub-cohort

Model 2 is further adjusted for education (no vocational training or in training, vocational training, technical school, technical college or university), occupation (sedentary, standing, (heavy) manual work), smoking behavior (never smoker, ex-smoker, current smoker <20 units/day, current smoker ≥20 units/day), sport activities (no sport, ≤4 h/week, >4 h/week), biking (no biking, <2.5 h/week, 2.5-4.9 h/week, ≥5 h/week), alcohol intake (women: no alcohol intake, >0-6 g/day, >6-12 g/day, >12 g/day; men: no alcohol intake, >0-6 g/day, >6-12 g/day, >12-24 g/day, >24 g/day), coffee consumption (ml/day), red meat intake (g/day), intake of whole-grain bread (g/day).
