# Supplementary material for: Association between the Fatty Liver Index and Risk of Type 2 Diabetes in the EPIC-Potsdam Study
Source: PLoS One. 2015 Apr 22;10(4):e0124749. doi: 10.1371/journal.pone.0124749 (PMC4406732; doi:10.1371/journal.pone.0124749)
Supplement: S2 Table — (DOCX) [file pone.0124749.s002.docx]

**S2_Table** HR (95% CI) for type 2 diabetes by categories of the fatty liver index in EPIC-Potsdam^a^ after exclusion of participants with heavy lifetime alcohol consumption (former heavy drinkers, occasionally heavy drinkers, always heavy drinkers)

|  | **FLI categories women** | | |  | **FLI categories men** | | |
| --- | --- | --- | --- | --- | --- | --- | --- |
|  | **<30** | **30-<60** | **≥60** |  | **<30** | **30-<60** | **≥60** |
| **FLI, median (IQR)^b^** | 7.17 (10.5) | 42.3 (14.7) | 77.1 (22.0) |  | 17.6 (13.6) | 44.6 (13.6) | 77.2 (18.3) |
| **n (cases)** | 38 | 69 | 129 |  | 15 | 52 | 230 |
| **Model 1**  (age-stratified) | 1 | 7.07  (4.49-11.1) | 16.1  (10.6-24.5) |  | 1 | 2.80  (1.50-5.23) | 11.0  (6.25-19.4) |
| **Model 2**  (multivariable-adjusted) | 1 | 7.10  (4.39-11.5) | 16.1  (10.1-25.6) |  | 1 | 2.37  (1.22-4.61) | 10.3  (5.65-18.7) |

FLI, fatty liver index

^a^ women: n=1346, men: n=850; ^b^ in sub-cohort

Model 2 is further adjusted for education (no vocational training or in training, vocational training, technical school, technical college or university), occupation (sedentary, standing, (heavy) manual work), smoking behavior (never smoker, ex-smoker, current smoker <20 units/day, current smoker ≥20 units/day), sport activities (no sport, ≤4 h/week, >4 h/week), biking (no biking, <2.5 h/week, 2.5- 4.9 h/week, ≥5 h/week), alcohol intake (no alcohol intake, >0-6 g/day, 6-12 g/day, >12-24 g/day, >24 g/day), coffee consumption (ml/day), red meat intake (g/day), intake of whole-grain bread (g/day).
