# Supplementary material for: Association between the Fatty Liver Index and Risk of Type 2 Diabetes in the EPIC-Potsdam Study
Source: PLoS One. 2015 Apr 22;10(4):e0124749. doi: 10.1371/journal.pone.0124749 (PMC4406732; doi:10.1371/journal.pone.0124749)
Supplement: S3 Table — (DOCX) [file pone.0124749.s003.docx]

**S3_Table** HR (95% CI) for type 2 diabetes in fasting participants by categories of the fatty liver index in EPIC-Potsdam^a^

|  | **FLI categories women** | | |  | **FLI categories men** | | |
| --- | --- | --- | --- | --- | --- | --- | --- |
|  | **<30** | **30-<60** | **≥60** |  | **<30** | **30-<60** | **≥60** |
| **FLI, median (IQR)^b^** | 7.01 (8.60) | 43.1 (16.5) | 76.6 (21.9) |  | 15.4 (11.1) | 43.9 (13.5) | 79.3 (18.1) |
| **n (cases)** | 14 | 20 | 42 |  | 6 | 23 | 86 |
| **Model 1**  (age-stratified) | 1 | 4.35  (2.12-8.94) | 12.2  (5.91-25.1) |  | 1 | 3.13  (1.24-7.89) | 11.3  (4.89-26.2) |
| **Model 2**  (multivariable-adjusted) | 1 | 4.74  (2.09-10.7) | 16.3  (6.79-39.1) |  | 1 | 2.52  (0.78-8.10) | 17.9  (6.82-47.0) |

FLI, fatty liver index

^a^ women: n=396, men: n=314; ^b^ in sub-cohort

Model 2 is further adjusted for education (no vocational training or in training, vocational training, technical school, technical college or university), occupation (sedentary, standing, (heavy) manual work), smoking behavior (never smoker, ex-smoker, current smoker <20 units/day, current smoker ≥20 units/day), sport activities (no sport, ≤4 h/week, >4 h/week), biking (no biking, <2.5 h/week, 2.5- 4.9 h/week, ≥5 h/week), alcohol intake (women: no alcohol intake, >0-6 g/day, >6-12 g/day, >12-24 g/day, >24-60 g/day, >60 g/day; men: no alcohol intake, >0-6 g/day, >6-12 g/day, >12-24 g/day, >24-60 g/day, >60-96 g/day, >96 g/day), coffee consumption (ml/day), red meat intake (g/day), intake of whole-grain bread (g/day).
