# Supplementary material for: Association between the Fatty Liver Index and Risk of Type 2 Diabetes in the EPIC-Potsdam Study
Source: PLoS One. 2015 Apr 22;10(4):e0124749. doi: 10.1371/journal.pone.0124749 (PMC4406732; doi:10.1371/journal.pone.0124749)
Supplement: S4 Table — (DOCX) [file pone.0124749.s004.docx]

**S4_Table** HR (95% CI) for type 2 diabetes in participants without prediabetes (HbA1c <5.7) by categories of the fatty liver index in EPIC-Potsdam^a^

|  | **FLI categories women** | | |  | **FLI categories men** | | |
| --- | --- | --- | --- | --- | --- | --- | --- |
|  | **<30** | **30-<60** | **≥60** |  | **<30** | **30-<60** | **≥60** |
| **FLI, median (IQR)^b^** | 7.08 (9.63) | 41.2 (15.4) | 73.9 (19.9) |  | 17.2 (14.0) | 43.7 (14.1) | 77.0 (19.3) |
| **n (cases)** | 20 | 23 | 33 |  | 8 | 24 | 64 |
| **Model 1**  (age-stratified) | 1 | 5.73  (3.02-10.9) | 13.6  (6.84-26.9) |  | 1 | 3.21  (1.37-7.53) | 7.12  (3.24-15.6) |
| **Model 2**  (multivariable-adjusted) | 1 | 6.39  (3.27-12.5) | 14.2  (6.64-30.3) |  | 1 | 2.88  (1.18-7.00) | 6.42  (2.78-14.9) |

FLI, fatty liver index

^a^ women: n=1023, men: n=600; ^b^ in sub-cohort

Model 2 is further adjusted for education (no vocational training or in training, vocational training, technical school, technical college or university), occupation (sedentary, standing, (heavy) manual work), smoking behavior (never smoker, ex-smoker, current smoker <20 units/day, current smoker ≥20 units/day), sport activities (no sport, ≤4 h/week, >4 h/week), biking (no biking, <2.5 h/week, 2.5- 4.9 h/week, ≥5 h/week), alcohol intake (women: no alcohol intake, >0-6 g/day, >6-12 g/day, >12-24 g/day, >24-60 g/day, >60 g/day; men: no alcohol intake, >0-6 g/day, >6-12 g/day, >12-24 g/day, >24-60 g/day, >60-96 g/day, >96 g/day), coffee consumption (ml/day), red meat intake (g/day), intake of whole-grain bread (g/day).
